# A Comprehensive Review on the Impact of Edible Coatings, Essential Oils, and Their Nano Formulations on Postharvest Decay Anthracnose of Avocados, Mangoes, and Papayas

**DOI:** 10.3389/fmicb.2021.711092

**Published:** 2021-07-30

**Authors:** Dharini Sivakumar, Nurdan Tuna Gunes, Gianfranco Romanazzi

**Affiliations:** ^1^Phytochemical Food Network, Department of Crop Sciences, Tshwane University of Technology, Pretoria, South Africa; ^2^Department of Horticulture, Faculty of Agriculture, Ankara University, Ankara, Turkey; ^3^Department of Agricultural, Food and Environmental Sciences, Marche Polytechnic University, Ancona, Italy

**Keywords:** chitosan, edible coatings, essential oil vapors, latent infections, biocontrol agents

## Abstract

Subtropical fruit such as avocados (*Persea americana*), mangoes (*Mangifera indica* L.), and papayas (*Carica papaya* L.) are economically important in international trade and predominantly exported to European destinations. These fruits are highly consumed due to their health benefits. However, due to long-distance shipping and the time required to reach the retail department stores, postharvest losses, due to postharvest decay occurring during the supply chain, affect the fruit quality on arrival at the long-distance distribution points. Currently, the use of synthetic fungicide, Prochloraz^®^, is used at the packing line to reduce postharvest decay and retain the overall quality of mangoes and avocados. Due to the ban imposed on the use of synthetic fungicides on fresh fruit, several studies have focused on the development of alternative technologies to retain the overall quality during marketing. Among the developed alternative technologies for commercial adoption is the use of edible coatings, such as chitosan biocontrol agents and essential oil vapors. The objective of this review is to summarize and analyze the recent advances and trends in the use of these alternative postharvest treatments on anthracnose decay in avocados, mangoes, and papayas.

## Introduction

Avocados are an economically important fruit crop in South Africa, Israel, the United States, Mexico, Peru, Chile, Kenya, and Australia. Papayas and mangoes from Latin American countries, especially from Brazil, are exported in large volumes to the EU. The demand for avocado fruit is rapidly increasing in the last few years due to its global recognition as a “superfruit,” based on nutritional facts and health benefits. The projection for the world avocado market by 2026 was to obtain an overall value of US$21.56 billion (Transparency Market Research Report, 2021). Consumption of avocados has increased in countries; the United States, and the EU and Latin American countries together totaled 1,323,000 tons in 2019 (Transparency Market Research Report, 2021). In 2019, the world export of papayas and mangoes was 2,575,002 and 364,030 tons, which fetched US$3,552,922 and US$295,628,000, respectively (FAOSTAT, 2021).

The export of fruits to the long-distance markets is via refrigerated sea shipment. The entire marketing time frame includes approximately 30 days until it reaches the consumers. During transport, due to cold storage and shelf life at the retail outlet, fruits develop postharvest decay caused by anthracnose (*Colletotrichum gloeosporioides* Penz and Sacc), which is the most common postharvest fungal disease in avocados (Sanders et al., 2000), mangoes (Lima J. R. et al., 2013), and papayas (Dickman, 1994). *C. gloeosporioides* infects the fruit via direct penetration, remains quiescent, and causes latent infections (Swinburne, 1983; Siddiqui and Ali, 2014). The latency was broken when the fruit reaches the climacteric peak (Swinburne, 1983). In the ripe fruit, anthracnose symptoms manifest as sunken, dark-brown to black decay lesions; and in the presence of high humidity, prominent pinkish-orange spore masses appear on the lesions (Nelson, 2008). Fruits infected by postharvest fungal pathogens cause decay symptoms, thus affecting the fruit quality, which negatively affects the market value. *C. gloeosporioides* reportedly infects at an immature stage of the fruit and lives quiescently (Beno-Moualem and Prusky, 2000). Postharvest loss of papayas was reportedly 30–50% due to anthracnose (Bautista-Baños et al., 2013). Avocadoes, mangoes, and papayas are climacteric fruits. Changes occurring during fruit ripening, and ethylene emission activate the infection process, which negatively affects their quality, shelf life, and market value. The previous review written by Sivakumar and Bautista-Baños (2014) highlighted the need for a natural novel fungicide to replace the synthetic fungicide application at the postharvest stage due to higher levels of pesticide residues in the edible portion of the fruit, pathogens developing resistance to fungicides, and the influence of fungicides on environmental footprint. However, still, the synthetic non-systemic fungicide Prochloraz^®^ {*N*-propyl-*N*-[2-(2,4,6-trichlorophenoxy) ethyl]-1*H*-imidazole-1-carboxamide} is applied at the packing line to control anthracnose decay in avocadoes, mangoes, and papayas (Swart et al., 2009; Henriod et al., 2016; Shimshoni et al., 2020). Although the exporting countries comply with the main trade regulations and the food safety requirements, the banning of Prochloraz^®^ fungicide will occur in 2022 due to its hazardous toxicological properties. The major metabolite of 2,4,6-trichlorophenol has been listed as a human carcinogen (Group B2) by the US Environmental Protection Agency (EPA) (Shimshoni et al., 2020; EPA, 2021). The “Farm to Fork Strategy” plays a major role in the European Green Deal set out to reduce reliance on pesticides, increasing organic farming and providing sustainable diets to benefit consumers’ health, which subsequently will help in reducing the medical care costs for treasury funds (EU COM, 2020).

Thus, investigations on numerous alternative postharvest treatments to control decay during the supply chain are ongoing to replace the application of Prochloraz^®^ fungicide at the postharvest stage. Furthermore, chloride-based sanitizers used in the packing line can produce carcinogenic compounds such as chloramines, dichloramines, and trichloromethanes, which have hazardous effects (Schoeny, 2010). The chemicals used in alternative treatments must have minimum toxicological effects on humans, domestic animals, and the environment and must fall under the category of generally recognized as safe (GRAS) by the US Food and Drug Administration (US FDA) and the European Food Safety Authority (EFSA). In addition, the alternative postharvest treatments must also be able to trigger induced defense mechanism of the host (fruit) via its eliciting properties and antimicrobial properties without compromising the fruit quality (Romanazzi et al., 2016). Considering the above, this review summarizes and analyzes the recent advances and trends in the use of these alternative postharvest treatments on the control of anthracnose caused by *C. gloeosporioides* in subtropical climacteric fruits during marketing based on published research articles from 2014 to date.

## Edible Coatings and Additives

Chitosan, a biodegradable compound, is a polymeric compound of *N*-acetylglucosamine units joined by *b*-1,4-glycosidic links. Its production is by the deacetylation of chitin through the alkaline hydrolysis process of acetamide groups using NaOH or KOH (3.7%) at a temperature of 71°C (Gómez-Ríos et al., 2017). The chitosan was registered in the EU as a basic substance for plant protection use (Reg. EU 662014/563), especially in the application for organic agriculture and integrated pest management. Romanazzi et al. (2018) highlighted the contribution of chitosan in the management of postharvest decay of fresh fruit and vegetables due to its antimicrobial, eliciting, and film-forming activities. Rajestary et al. (2021) ran meta-analyses in which it were summarized as clear antimicrobial activity and decay control, while eliciting activity was often clarified and varied among the different studies for time and involved compounds. Romanazzi et al. (2018) showed a list of some of the available chitosan-based commercial products in countries such as Germany, the United States, the United Kingdom, Chile, New Zealand, Italy, Thailand, Poland, and China. Factors such as molecular weight of chitosan formulation, its concentration, method of application, and storage temperature of the fruit affect the efficacy of chitosan application on control of fruit decay (Velázquez-Del Valle et al., 2012). Marques et al. (2016) reported that chitosan, at concentrations of 1.0, 1.5, and 2.0%, inhibited the mycelial growth of *C. gloeosporioides* by 75, 86, and 90%, respectively; and at higher concentrations, 2.5 and 3.0%, showed a fungicidal effect by resulting in changes in conidial morphology. Marques et al. (2016) further reported that chitosan at concentrations of 1.5 and 2.0% completely arrested the germ tube formation and the germination process in *C. gloeosporioides*. Controlling the mycelial growth and the germination of *C. gloeosporioides* by chitosan demonstrates its fungicide potential. The electrostatic forces between its amino-protonated (NH^2+^) groups and negative residues on cell surfaces of *C. gloeosporioides* (pathogen) were responsible for the observed antifungal activity facilitated by chitosan (Elsabee and Abdou, 2013; Aloui et al., 2014). Commercial chitosan application (ChitoPlant^®^) at 1.5%, the formulation that dissolves in water, decreased anthracnose rot in avocados during a storage period of 28 days and after 5 days at marketing simulation conditions (Obianom et al., 2019). The mode of mechanism was attributed to the upregulation of phenylalanine ammonia-lyase, downregulation of lipoxygenase genes, and moderate retention of epicatechin content (90 mg kg^–1^ FW) in the skin probably due to delayed ripening and ethylene emission that had reduced the anthracnose decay (Obianom et al., 2019). Control of stem-end rot decay in commercial chitosan-coated (ChitoPlant^®^ 1.5%) fruit was due to the upregulation of chitinase genes and higher superoxide dismutase activity (Obianom et al., 2019). Moreover, the non-toxic and antimicrobial properties and biodegradable nature of chitosan made it the most important natural biopolymer material for agriculture biotechnology. Conversely, avocados coated with carboxyl methylcellulose (1%) containing 2% of moringa leaf extract controlled the anthracnose decay by inhibiting the mycelial growth of *C. gloeosporioides* and maintaining the overall quality and shelf life of the fruits during marketing (Tesfay et al., 2017). Moringa leaf extract incorporated into carboxymethyl cellulose, exposed to gaseous ozone (O_3_) treatments for 36 h and thereafter, stored at 10°C and 95% relative humidity (RH) for 21 days, extended the shelf life, and maintained the fruit quality of mangoes fruit (‘Keitt’) by reducing the decay (Bambalele et al., 2021). Avocado ‘Maluma Hass’ fruit treated with 1% carboxyl methylcellulose + 10% moringa extract maintained the postharvest quality of fruit stored for 3 weeks at 5.5°C and at the simulated retail shelf conditions for an additional 1 week (Kubheka et al., 2021). Fruits coated with edible coatings form a semi-permeable layer around the fruit and create a modified atmosphere (Ali et al., 2014), and this modifies the internal atmosphere of the fruit, which helped to reduce the decay incidence and severity as compared with the control fruits (Garcia and Davidov-Pardo, 2021). Modified atmospheres created by the coating around the fruit could have reduced the pH (lower) of the skin, which is unfavorable to decay-causing *C. gloeosporioides* (Yakoby et al., 2000). Ali et al. (2014) recommended the use of the ethanolic extract of propolis (1.5%) and gum arabic (10%) as a biofungicide for the control of anthracnose in papayas. In addition, the edible coatings reduce the rate of respiration, moisture loss, senescence, and weight loss due to the semi-permeable modified atmosphere layer on the fruit surface (Ali et al., 2014). Table 1 summarizes the recent advances on edible coating and additives applied for subtropical fruits avocadoes and mangoes.

**TABLE 1:** Recent advances on edible coating and additives applied for subtropical fruits avocado and mangoes.

**Edible coatings**	**Findings and mode of action**	**References**
Chitosan at concentrations 2.5 and 3.0%	Higher concentrations 2.5 and 3.0% showed fungicidal effect by resulting in changes in conidial morphology of *C. gloeosporioides*	Marques et al., 2016
Commercial chitosan application (ChitoPlant^®^) at 1.5% concentration	Up-regulation of phenylalanine ammonia-lyase and down-regulation of lipoxygenase genes and moderate retention of epicatechin content in avocados (‘Hass’)	Obianom et al., 2019
Carboxyl methylcellulose (1%) containing 2% ethanolic moringa leaf extract	Mycelial growth of *C. gloeosporioides*	Tesfay et al., 2017
Carboxyl methylcellulose (1%) containing 2% ethanolic moringa leaf extract, exposed to (O_3_) before storage	Reduced the fruit decay and improved fruit quality of mango (‘Keitt’). Exact mechanism not known	Bambalele et al., 2021
1% carboxyl methylcellulose + 10% moringa extract	Reduced the fruit decay and improved fruit quality of avocado Maluma Hass. Probably due to modification of the internal atmosphere causing the maintenance of a lower pH of the skin, which is unfavorable to *C. gloeosporioides* (Yakoby et al., 2000)	Yakoby et al., 2000; Garcia and Davidov-Pardo, 2021; Kubheka et al., 2021

## Nanomaterial Antifungal Properties

Nanoparticles are considered effective fungicides to control fungal pathogens in crops due to their distinctive physical and chemical properties, which do not relate to their bulk properties (De la Rosa-García et al., 2018). The antifungal effects of the nanoparticles are due to the alternation of physicochemical properties (optical, catalytic, and electronic properties) associated with size (De la Rosa-García et al., 2018). Nanoparticles of zinc oxide (ZnO) were evaluated against *C. gloeosporioides* obtained from papayas and avocadoes (De la Rosa-García et al., 2018). ZnO nanoparticles inhibited the fungal growth of *C. gloeosporioides in vitro* by causing structural deformation by vacuolar expansion, swelling, and melanization in the spores and mycelia (De la Rosa-García et al., 2018). The mode of action of ZnO nanoparticles is due to the production of reactive oxygen species (ROS) in water suspensions (Applerot et al., 2010). ZnO is listed as GRAS by the US FDA (21CFR182.8991) (FDA, 2021). However, further toxicological studies on the migration of nanoparticles into the fruit and toxicological studies are required to ensure that fruits coated with nanoparticle formulations are safe for consumption.

## Nanomaterials and Nano-Chitosan Coating

Research on the incorporation of nanosized edible coatings, such as chitosan, with natural antimicrobials, such as essential oils, to improve fruit health, safety, and shelf life has become popular during the past 5 years. Nanosized chitosan particles are non-toxic, biodegradable, highly permeable through the biological membranes, and cost-effective and have a wide spectrum of antifungal activities, therefore regarded as a suitable natural agent for control of postharvest decay (Meena et al., 2020).

Chitosan coating with silver nanoparticle (chitosan-AgNP), having the size distribution from 10 to 15 nm, significantly inhibited the conidial germination and inhibited the anthracnose decay significantly in mango fruits. Incorporating the silver nanoparticle to chitosan formulation aided in the binding interaction and stabilization by helping the dispersion of the chitosan-AgNP composites in the formulation during application to improve the efficacy (Chowdappa et al., 2014).

However, the EU does not permit food products or packaging that possesses silver nanoparticles (AgNPs) without any authorization. Allowable limits, based on the EFSA (EFSA, 2011), must not exceed 0.05 mg L^–1^ in water and 0.05 mg kg^–1^ in food. This emphasizes that the testing for antimicrobial effectiveness should comply with the current regulations. Furthermore, incorporations of antimicrobials, such as essential oils, in edible coatings or films are commercially adoptable due to the cost-effectiveness of low volumes of essential oils, effective diffusion efficiency, and prolonged antimicrobial activity during marketing.

Within essential oils, the US FDA Regulatory Agency (FDA, 2021) considers thyme oil (TO) as GRAS. Nanostructured edible coatings based on chitosan (3%)–thyme essential oil (5%) nanoparticles showed a synergetic effect on the control of anthracnose in avocadoes (cv. Hass) by arresting the growth of *C. gloeosporioides* (Correa-Pacheco et al., 2017). The drawback of this study was that there was no comparison of the efficacy of the antifungal activity of chitosan nanoparticles-essential oil coating with the currently used commercial Prochloraz^®^ fungicide, and it was not tested on the avocado cultivar Fuerte, which is highly susceptible to anthracnose decay. In addition, nano or microencapsulation of essential oils prevents the degradation of the active compounds and their stability during unfavorable environmental conditions, such as heat, oxygen, light, pressure, moisture, and pH (Majeed et al., 2015).

The efficacy of the chitosan nanoparticles-essential oil coating also depends on the release rate and migration of active components of essential oils from the edible coating matrix. Additional investigations are required on the influence of incorporation of chitosan nano formulation-TO on fruit quality by measuring the gas exchange barrier, water vapor transmission, tensile and transparent properties of the coatings, internal and external fruit quality, and organoleptic characteristics (Flores-López et al., 2016). Chitosan nanoparticle-essential oil coatings can affect the ripening pattern, flesh color, weight loss, fruit firmness, and degradation of phenolic compounds and antioxidant property of the subtropical fruits avocadoes, papayas, and mangoes. Furthermore, the inclusion of essential oils inside of chitosan nanoparticles helps in releasing and reducing the active compounds (Mohammadi et al., 2015).

In addition, chitosan nanomaterials contain functionalizing agents, such as Cu, Zn, and salicylic acid (SA), which were added to improve the upregulation of antioxidant-defense enzymes in the plant cells, regulate the plant cellular homeostasis, and provide protection against diseases (Saharan et al., 2015; Kumaraswamy et al., 2019). The Cu–chitosan nanoparticles were tested for postharvest application (Meena et al., 2020). The mode of action was due to induced protonation of amino groups of chitosan because of acidic pH that released Cu ions from chitosan nanostructures to perform the antifungal activity. The Cu^2+^ ions showed an antifungal activity by improving the production of highly reactive hydroxyl radicals that eventually harm the biomolecules of the fungal cells (Meena et al., 2020). Chitosan/nano-silica coating enhanced the activities of antioxidant enzymes (superoxide dismutase, catalase, and ascorbate peroxidase) and reduced the generation of superoxide anion (O^2–^) and hydrogen peroxide (H_2_O_2_) (Song et al., 2016). Nitric oxide-releasing chitosan nanoparticles also reduced ethylene production and ROS and increased the antioxidant enzyme activity and antioxidant capacity in fruits (Ma et al., 2019).

Biopolymer chitosan is a suitable alternative to synthetic polymeric materials due to its biodegradable nature and negative impact on the environment. Chitosan and TiO_2_ nanocomposite films maintained the quality of climacteric fruit by photodegradation of ethylene activity in the presence of UV light, which caused delayed fruit ripening (Kaewklin et al., 2018). Furthermore, a combination of 3% CaCl_2_ and nano-chitosan improved the antioxidant capacity by improving the hydroxyl radical (•OH) scavenging capacity and possibly could have acted as “signalling compounds” triggering the increase in antioxidant activities (enzymatic and non-enzymatic) in tissues (Jin et al., 2012; Nguyen et al., 2020). Table 2 summarizes the recent developments in nanomaterial antifungal properties.

**TABLE 2:** Summary of recent developments in nano materials antifungal properties.

**Nano materials**	**Findings and mode of action**	**References**
Nanoparticles of zinc oxide (ZnO)	Production of oxygen species (ROS) in water suspensions. Structural deformation by vacuolar expansion, swelling, and melanization in the spores and mycelia of *C. gloeosporioides*	Applerot et al., 2010; De la Rosa-García et al., 2018
Chitosan coating with silver nanoparticle (AgNP)	Incorporation of silver nanoparticle to chitosan formulation helped in binding interaction and stabilization to help dispersion of the chitosan-AgNP composites in the formulation improve the efficacy. Inhibited the conidial germination	Chowdappa et al., 2014
Chitosan-thyme essential oil nanoparticles	Chitosan nanoparticles at 3% and thyme oil at 5% showed fungicidal effect and reduced the anthracnose decay fruit cv. Hass	Correa-Pacheco et al., 2017
Cu–chitosan nanoparticles	The Cu^2+^ ions showed antifungal activity by improving the production of highly reactive hydroxyl radicals that eventually harm the biomolecules of the fungal cells	Meena et al., 2020
Chitosan/nano-silica coating	Enhanced the activities of antioxidant enzymes (superoxide dismutase, catalase, and ascorbate peroxidase) and reduced the generation of superoxide anion (O^2–^) and hydrogen peroxide (H_2_O_2_)	Song et al., 2016
Nitric oxide-releasing chitosan nanoparticles	Reduced the ethylene production, reactive oxygen species, and increased the antioxidant enzyme activity and antioxidant capacity in fruits	Ma et al., 2019
Chitosan and TiO_2_ nanocomposite films	Maintained the quality of climacteric fruit by photodegradation of ethylene activity in presence of UV light, which caused delayed fruit ripening	Kaewklin et al., 2018
Combination of 3% CaCl_2_ and nano-chitosan	Chitosan improved the antioxidant capacity by improving the hydroxyl radical (∙OH) scavenging capacity, and possibly could have acted as “signaling compounds” triggering the increase in increases in antioxidant activities (enzymatic and non-enzymatic) in tissues	Nguyen et al., 2020; Jin et al., 2017
Nano emulsions of orange essential oil (*Citrus sinensis*) and Xoconostle (*Opuntia oligacantha*) extract	Improved the antioxidant activity due to the signaling effect of active compounds of essential oils	Cenobio-Galindo et al., 2019

## Essential Oils

Most essential oils are under the category of GRAS by the US FDA (FDA, 2021). Nanoemulsions of orange essential oil (*Citrus sinensis*) and xoconostle (*Opuntia oligacantha*) extract controlled anthracnose, increased the retention of fruit firmness in avocados, and improved the antioxidant activity due to the signaling effect of the active compounds of essential oils (Cenobio-Galindo et al., 2019). Furthermore, basil oil incorporated with beeswax coating increased shelf life and reduced anthracnose development in mango cv. Willard (Karunanayake et al., 2020). Rosemary pepper and Noni essential oils effectively controlled the *C. gloeosporioides* isolated from the papaya fruit (Dias et al., 2020). Noni essential oils totally inhibited conidial germination at 3,000 ppm, while rosemary pepper inhibited conidial germination at 5,000 ppm. The inhibition of mycelial growth of *C. gloeosporioides* occurred at 4,000- and 6,000-ppm concentrations with Noni and rosemary pepper essential oils (Dias et al., 2020). Savory oil with the active chemical compound carvacrol (71.2%) completely controlled the mycelial growth of *C. gloeosporioides* obtained from the papaya fruit (Sarkosh et al., 2018). *C. gloeosporioides* isolated from mangoes was effectively controlled by thymol-based essential oil (Chillet et al., 2018). Essential oils also cause structural and functional damage in the microbial cell by affecting the membrane permeability, causing leakage of cellular constituents, and dissipating the H^+^ and K^+^ ion gradients (Sarkosh et al., 2018).

Obianom and Sivakumar (2018) illustrated that the incorporation of 0.1% (v/v) TO in half-strength Prochloraz^®^ (reduced concentration) completely reduced the anthracnose rot in avocado cv. Fuerte. The incorporation of TO had induced the activity of defense enzymes and resulted in decay reduction (Obianom and Sivakumar, 2018). The growers prefer the TO application in the aqueous phase, as it is easy to implement on the packing line.

Spray-dried TO microencapsulated with modified starch/agave fructan microcapsules, 0.10 and 0.20 g filled in nylon bags (4 × 4 cm) (antifungal sachets), controlled the mycelial growth of *C. gloeosporioides* and reduced the anthracnose incidence in mangoes during storage in boxes, which were sealed with parafilm for 9 days at 20 ± 2°C (Esquivel-Chávez et al., 2021). Active components of TO together triggered the induced defense mechanism by upregulation of phenylalanine ammonia-lyase, chitinase, and β-1,3-glucanase enzymes in the fruit and exocarp while downregulating the lipoxygenase enzymes, which helped to retain a moderately higher concentration of epicatechin on the exocarp and delayed the fruit ripening (Bill et al., 2017). Furthermore, *C. gloeosporioides* isolated from the papaya fruit (‘Sekaki’) exposed to lemongrass oil vapor at a concentration of 28 μL L^–1^ containing geranial (45.6%) and neral (34.3%) as the major components for 18 h and stored for 9 days controlled the anthracnose decay due to fungicidal properties (Ali et al., 2015). Table 3 summarizes the recent developments in application of essential oils volatile components on the control of postharvest decay in avocado, mango and papaya. In addition, the starch film incorporated with carvacrol (750 mg L^–1^) and thymol (750 mg L^–1^) in the starch film showed complete inhibition of *C. gloeosporioides* (*in vitro*) (Ochoa-Velasco et al., 2021). The synergetic effect of the chemical constituents of essential oil components is due to the increased phenolic compounds in the molecule structure and could have facilitated the binding of these compounds to the proteins, preferably the cell membrane of the pathogens, which could have facilitated the membrane disintegration and loss of cellular content (Ochoa-Velasco et al., 2021). Furthermore, an antimicrobial transparent flexible trilayer low-density polyethylene (LDPE) film containing organically modified layered double hydroxides (OLDHs) and plant bioactive TOTO showed a remarkable reduction in anthracnose disease events in Hass cultivar (Kesavan Pillai et al., 2020). The barrier and antifungal properties of nanocomposite film helped to release the volatile bioactive release in a controlled manner; thus, the synergistic effect of LDPE–OLDH–TO nanocomposite film can act as a prospective strategy to control anthracnose disease in avocados with modification of headspace gas composition (Kesavan Pillai et al., 2020).

**TABLE 3:** Summary of recent developments in essential oils and incorporation of essential oils into wax or films on antifungal properties against *C. gloeosporioides*.

**Essential oils**	**Findings and mode of action**	**References**
Nano emulsions of orange essential oil (*Citrus sinensis*) and xoconostle *(Opuntia oligacantha)* extract	Controlled anthracnose, increased the retention of fruit firmness in avocados, and improved the antioxidant activity due to the signaling effect of the active compounds of essential oils.	Cenobio-Galindo et al., 2019
Basil oil incorporated with beeswax coating	Increased shelf life and reduced anthracnose development in mango cv. ‘Willard.’	Karunanayake et al., 2020
Rosemary pepper and Noni essential oils	Controlled the *C. gloeosporioides* isolated from papaya fruit by inhibiting the conidial germination.	Dias et al., 2020
Savory oil with active chemical compound carvacrol (71.2%)	Controlled the mycelial growth of *C*. *gloeosporioides* obtained from papaya fruit. Essential oils caused structural and functional damage in the microbial cell by affecting the membrane permeability, causing leakage of cellular constituents, and dissipating the H^+^ and K^+^ ion gradients.	Sarkosh et al., 2018
Incorporation of 0.1% (v/v) thyme oil in half-strength Prochloraz^®^ (reduced concentration)	Completely reduced the anthracnose rot in avocado cv. ‘Fuerte.’ The incorporation of thyme oil had induced the activity of defense enzymes and resulted in decay reduction.	Obianom and Sivakumar, 2018
Spray-dried thyme oil microencapsulated with modified starch/agave fructan microcapsules, 0.10 and 0.20 g filled in nylon bags (4 × 4 cm) (antifungal sachets)	Controlled the mycelial growth of *C. gloeosporioides* and reduced the anthracnose incidence in mangoes. Active components of thyme oil together triggered the induced defense mechanism; upregulation of phenylalanine ammonia-lyase, chitinase, and β-1,3-glucanase enzymes in the fruit and exocarp, while downregulating the lipoxygenase enzymes, moderately higher concentration of epicatechin on the exocarp; delayed fruit ripening.	Bill et al., 2017; Esquivel-Chávez et al., 2021
*C. gloeosporioides* isolated from papaya fruit (‘Sekaki’) exposed to lemon grass oil vapor at concentration of 28 μg L^–1^ containing geranial (45.6%) and neral (34.3%) as the major components for 18 h and stored for 9 days	Controlled the anthracnose decay due to fungicidal properties.	Ali et al., 2015
Starch film incorporated with carvacrol (750 mg L^–^1) and thymol (750 mg L^–^1)	Inhibition of *C. gloeosporioides* (*in vitro*). The synergetic effect of the chemical constituents of essential oil components; due to the increased phenolic compounds in the molecule structure; membrane disintegration and loss of cellular content.	Ochoa-Velasco et al., 2021
Flexible trilayer low density polyethylene (LDPE) film containing organically modified layered double hydroxides (OLDH) and plant bioactive thyme oil (TO)	Reduction in anthracnose disease events in ‘Hass’ cultivar. The barrier and antifungal properties of nanocomposite film helped to release the volatile bioactive release in a controlled manner.	Kesavan Pillai et al., 2020

## Biocontrol

The use of biocontrol agents is for the managing of postharvest decay of fruit as an alternative treatment to synthetic fungicides. The ideal antagonist should be able to survive adverse environmental conditions, should use an affordable growth substrate for mass production, should not infect the host plant, or should not produce metabolites that are toxic for consumers; it must be resistant to the commonly used pesticides and well suited with other chemical and physical postharvest treatments (Wilson and Wisniewski, 1989). The efficiency of biocontrol products could be improved during an integrated control strategy to reduce fungicide input (Govender et al., 2005). Biocontrol products, Biosave-100 and Biosave-110, were commercially used as a postharvest treatment especially for apples: Bio-Save [biocontrol agent (b.a) *Pseudomonas syringae*], Boni Protect (b.a *Aureobasidium pullulans*), Candifruit (b.a *Candida sake*), Nexy (b.a *Candida oleophila*), Pantovital (b.a *Pantoea agglomerans*), Shemer (b.a *Metschnikowia fructicola*), and Yield Plus (b.a i. *Cryptococcus albidus*) (Sharma et al., 2009; Spadaro and Droby, 2016; Janisiewicz and Jurick, 2017). However, commercial biocontrol products are limited to the control of anthracnose decay in avocados. Biocontrol agent *Debaryomyces nepalensis* completely controlled the anthracnose decay in mangoes at 1×10^7^ cells mL^–1^ concentration at 15°C for 30 and 40 days (Konsue et al., 2020). Postharvest treatment with *D. nepalensis* reduced the anthracnose decay by delaying the ripening and senesce of the fruit caused due to an increase in cell membrane permeability and less lipid peroxidation (malondialdehyde content) than the control during storage (Konsue et al., 2020). The yeast, *Torulaspora indica* DMKU-RP31, controlled the controlling anthracnose and stem-end rot in mango fruits, and the volatiles produced by the yeast strain were responsible for the antifungal property (Konsue et al., 2020). Furthermore, dipping of the mangoes in *Metschnikowia pulcherrima* suspension (1.0 × 10^8^ cfu mL^–1^), SA solution (50 mg L^–1^), or calcium chloride (CaCl_2_) solution (1.0 g L^–1^) reduced the anthracnose symptoms in fruit by inducing the defense-related enzymes phenylalanine ammonia-lyase, chitinase, and β-1,3-glucanase compared with the control treatment (Tian et al., 2018). Yeast *Meyerozyma caribbica* in sodium alginate coating controlled the anthracnose (*C. gloeosporioides* Pa14) in avocado fruit (Iñiguez-Moreno et al., 2020). The sodium alginate coating helped as a matrix to trap the yeast and the volatile compounds, especially 1-butanol, 3-methyl- and phenethyl alcohol, and ethyl acetate, to arrest the invasion of the pathogen (Iñiguez-Moreno et al., 2020). The alcohols cause destruction of plasma membrane due to accelerated degeneration of protein molecules that eventually affect the metabolism and cell lysis (McDonnell and Russell, 1999). With ripening, the yeast population increased on the fruit surface and provided residual protection against anthracnose (Iñiguez-Moreno et al., 2020). Besides, *M. caribbica* also showed the mode of action due to competition for nutrients, biofilm development, and production of β-1,3-glucanase and chitinase enzymes (Iñiguez-Moreno et al., 2020).

Furthermore, cell-free supernatant of *Bacillus atrophaeus* strain B5 inhibited the mycelial growth and conidial germination of *C. gloeosporioides in vitro* and reduced the severity and incidence of anthracnose disease on avocado fruit. *B. atrophaeus* strain B5 harbors a gene that expressed the biosynthesis of antibiotics, mainly surfactin, bacillomycin, and iturin, which probably could have shown antifungal activities against *C. gloeosporioides* (Guardado-Valdivia et al., 2018). Inhibition of spore germination is vital to control the inoculum load at an early stage of decay development. The lipopeptides fengycins and iturins showed a higher antifungal activity than surfactin (Ongena and Jacques, 2008; Meena and Kanwar, 2015). Some lipopeptides (surfactin and other cyclic lipopeptides) were shown to elicit induced systemic resistance (ISR) in the host (Ongena and Jacques, 2008; Raaijmakers et al., 2010; Gond et al., 2015). Since the biocontrol agents do not fall under the category of GRAS compounds, the metabolites in the cell-free supernatants can be of great interest for the control of postharvest decay in avocado, mango, or papaya fruits (Guardado-Valdivia et al., 2018).

Marine yeast *Debaryomyces hansenii* 1R11CB strain and marine bacteria *Stenotrophomonas rhizophila* KM02 strain were identified as the suitable antagonistic agents to anthracnose (*C. gloeosporioides*) in mangoes (‘Ataulfo’) and reduced the decay incidence to 56 and 89%, respectively (Hernandez Montiel et al., 2017). The authors suggest that the possible mechanisms could be (i) antagonists competing for nutrients such as glucose, sucrose, and fructose with *C. gloeosporioides* due to their rapid growth rate, which affects the infection process of the pathogen by colonizing the hyphae of *Colletotrichum*; or (ii) by secreting chitinase to degrade β-glucan, mannoprotein, and chitin, respectively, of the cell wall components of *C. gloeosporioides* (Vero et al., 2013; Liu et al., 2016); or (iii) by inducing the host defense mechanism by triggering the antioxidants, peroxidase, catalase, and superoxide dismutase and of disease-defense enzyme phenylalanine ammonia-lyase, chitinase, and β-1,3-glucanase indirectly and preventing the entry of *C. gloeosporioides* (Wei et al., 2016; Wu et al., 2019). Furthermore, yeast *Wickerhamomyces anomalus* reduced the anthracnose (*C. gloeosporioides*) incidence in avocado fruit and provided effective protection against anthracnose (Campos-Martínez et al., 2016). *W. anomalus* showed mycoparasitism with *C. gloeosporioides* hyphae and caused the loss of turgor pressure and eventually yeast gaining entry into the fungal cells via cell walls (Lima N. B. et al., 2013; Campos-Martínez et al., 2016). Table 4 summarizes the recent developments in biocontrol agent with antifungal properties against *C. gloeosporioides* infecting mangoes and avocados.

**TABLE 4:** Summary of recent developments in biocontrol agent with antifungal properties against *Colletotrichum gloeosporioides* infecting mango and avocados.

**Biocontrol agents**	**Findings**	**Mode of action**	**References**
Yeast, *Torulaspora indica* DMKU-RP31	Controlled anthracnose *in* mango fruits	Antifungal volatiles but the components of volatiles not detected	Konsue et al., 2020
Combination of *Metschnikowia pulcherrima* suspension in, salicylic acid (SA) solution (50 mg L^–1^), or calcium chloride (CaCl_2_) solution (1 g L^–1^)	Reduced the anthracnose symptoms in the mango fruit	Inducing the defense-related enzymes phenylalanine ammonia-lyase, chitinase, and β-1,3-glucanase	Tian et al., 2018
*Meyerozyma caribbica* in sodium alginate coating	Controlled the anthracnose (*C. gloeosporioides* Pa14) in avocado fruit	Sodium alginate coating helped as a matrix to trap the yeast and the volatile compounds 1-butanol, 3-methyl- and phenethyl alcohol, and ethyl acetate to arrest the invasion of the pathogen	Iñiguez-Moreno et al., 2020
Cell free supernatant of *Bacillus atrophaeus* strain B5 harbors	Inhibited the mycelial growth and conidial germination of *C. gloeosporioides in vitro* and also reduced severity and incidence of anthracnose disease on avocado fruit	Expressed the biosynthesis of antibiotics mainly surfactin, bacillomycin, and iturin and probably could have shown antifungal activities against *C. gloeosporioides*	Guardado-Valdivia et al., 2018
Marine yeast *Debaryomyces hansenii* 1R11CB strain and marine bacteria *Stenotrophomonas rhizophila* KM02 strain	Reduced the decay incidence in mango (‘Ataulfo’)	Antagonists competing for nutrients such as glucose, sucrose, and fructose, colonizing the hyphae of *Colletotrichum* hyphae	Hernandez Montiel et al., 2017
*Wickerhamomyces anomalus*	Reduced the anthracnose incidence in avocado fruit	Mycoparasitism with *C. gloeosporioides* hyphae	Lima N. B. et al., 2013; Campos-Martínez et al., 2016

## Concluding Remarks

Alternative treatments, edible coatings, films and additives, nanomaterials, essential oils, and biocontrol agents could be more effective during integrated combination via a “hurdle concept” due to their different mechanisms. The alternative products replacing the commercial synthetic fungicides should not affect the fruit quality or sensory properties of the fruit. In addition, during the application, the product should not affect the environment and other organisms that are agriculturally friendly. Furthermore, the practical application, reliability of the product, commercialization, registration, and cost-effectiveness of the treatments are important before recommending the treatments to the avocado, mango, and papaya industries.

## Author Contributions

All authors listed have made a substantial, direct and intellectual contribution to the work, and approved it for publication.

## Conflict of Interest

The authors declare that the research was conducted in the absence of any commercial or financial relationships that could be construed as a potential conflict of interest.

## Publisher’s Note

All claims expressed in this article are solely those of the authors and do not necessarily represent those of their affiliated organizations, or those of the publisher, the editors and the reviewers. Any product that may be evaluated in this article, or claim that may be made by its manufacturer, is not guaranteed or endorsed by the publisher.
